# Gellan-Based Hydrogel as a Drug Delivery System for Caffeic Acid Phenethyl Ester in the Treatment of Oral *Candida albicans* Infections

**DOI:** 10.3390/pharmaceutics16030298

**Published:** 2024-02-20

**Authors:** Maíra Terra Garcia, Paulo Henrique Fonseca do Carmo, Lívia Mara Alves Figueiredo-Godoi, Natália Inês Gonçalves, Patrícia Michelle Nagai de Lima, Lucas de Paula Ramos, Luciane Dias de Oliveira, Alexandre Luiz Souto Borges, Anita Shukla, Juliana Campos Junqueira

**Affiliations:** 1 Department of Biosciences and Oral Diagnosis, Institute of Science and Technology, São Paulo State University (UNESP), São José dos Campos 12245-000, SP, Brazil; 2 Department of Dental Materials and Prosthodontics, Institute of Science and Technology, São Paulo State University (UNESP), São José dos Campos 12245-000, SP, Brazil; 3 Center for Biomedical Engineering, School of Engineering, Brown University, Providence, RI 02912, USA

**Keywords:** *Candida albicans*, oral candidiasis, denture stomatitis, gellan gum, caffeic acid phenethyl ester, antifungal

## Abstract

*Candida albicans* can cause various types of oral infections, mainly associated with denture stomatitis. Conventional therapy has been linked to high recurrence, toxicity, and fungal resistance, necessitating the search for new drugs and delivery systems. In this study, caffeic acid phenethyl ester (CAPE) and gellan gum (GG) were studied as an antifungal agent and carrier system, respectively. First, we observed that different GG formulations (0.6 to 1.0% wt/vol) were able to incorporate and release CAPE, reaching a controlled and prolonged release over 180 min at 1.0% of GG. CAPE-GG formulations exhibited antifungal activity at CAPE concentrations ranging from 128 to >512 µg/mL. Furthermore, CAPE-GG formulations significantly decreased the fungal viability of *C. albicans* biofilms at short times (12 h), mainly at 1.0% of GG (*p* < 0.001). *C. albicans* protease activity was also reduced after 12 h of treatment with CAPE-GG formulations (*p* < 0.001). Importantly, CAPE was not cytotoxic to human keratinocytes, and CAPE-GG formulations at 1.0% decreased the fungal burden (*p* = 0.0087) and suppressed inflammation in a rat model of denture stomatitis. Altogether, these results indicate that GG is a promising delivery system for CAPE, showing effective activity against *C. albicans* and potential to be used in the treatment of denture stomatitis.

## 1. Introduction

*Candida albicans* is the chief pathogen of mucosal and systemic candidiasis worldwide, and is associated with high rates of mortality, morbidity, and a considerable economic burden on the healthcare system [1,2,3]. In the dental area, *C. albicans* is a major concern due to its ability to cause oral infections in the presence of various predisposing factors, such as denture stomatitis, which affects most of the denture wearing population. Oral candidiasis is an opportunistic infection caused by yeast overgrowth and penetration of hyphae and pseudohyphae into oral tissues. In *C. albicans*-associated denture stomatitis, yeast overgrowth occurs on the denture surface [4]. In this process, several virulence factors of *C. albicans* are important, such as surface adhesins expression, the yeast-to-hypha transition, biofilm formation, and the production of enzymes, mainly secreted aspartyl proteases (saps) [5,6].

In immunocompromised and elderly patients, oral candidiasis and denture stomatitis are difficult to treat, and recurrence of infection becomes very frequent [7]. Furthermore, the denture acts as a reservoir, enabling *C. albicans* to re-infect the mucosal surface continually and increasing the risk of candidemia [4,8]. Conventional therapy of oral candidiasis and denture stomatitis involves mainly antifungal agents, such as nystatin, miconazole, and fluconazole. However, the treatment has been associated with transient improvement, high recurrence, toxicity, and fungal resistance [2,9]. For this purpose, several alternative therapies have been studied, including the development of new drugs, and drug delivery systems [10,11,12].

In this scenario, caffeic acid phenethyl ester (CAPE), a bioactive compound derived from propolis, was studied as a possible antifungal agent. Previous studies demonstrated its anti-inflammatory [13], antioxidant [14], antimicrobial [15] and immunomodulatory effects [16]. In addition, CAPE has shown synergistic effects with fluconazole, nystatin, and caspofungin against *C. albicans* [17,18]. In our previous study, CAPE showed in vitro antifungal activity against *C. albicans* and efficacy to treat oral candidiasis in mice [15]. However, the CAPE solution has limited clinical application due to its low viscosity and short retention time in the oral cavity, which demands its incorporation in formulations and carrier systems.

Various biomaterials exhibit promising activity as drug delivery systems due to their ability to release controlled and prolonged concentrations of the drug at the site of infection, minimizing the risk of fungal resistance, relapses, and systemic toxicity [19,20,21,22]. Among these biomaterials, gellan-based hydrogels have been employed for local delivery of drugs or probiotics, targeting for ophthalmic, vaginal, nasal, and oral infections [10,23,24,25,26,27]. Gellan gum (GG) is a water-soluble anionic exopolysaccharide produced by the bacterial genus *Sphingomonas,* which gels in the presence of cations [28]. Its polymer chain consists of a tetrasaccharide repeating unit of L-rhamnose, D-glucose and D-glucuronate. Gellan gum has an average molecular weight around 500 kDa and can tolerate heat and acid stress during the fabrication process [29].

GG is approved by the Food and Drug Administration (FDA) as a gelling agent in the food. Recently, GG has drawn attention to pharmaceutical and medical industries due to its functional properties that includes: stability after heating or pH variations, dispersibility, and versatile texture, which allows the adjustment of hardness, firmness, brittleness, and elasticity. GG is also environmentally friendly, sustainable, and inexpensive [21,30,31]. In addition, previous studies showed that carrier systems using GG had good biocompatibility with oral tissues and protected the incorporated drug against the enzymatic activity of saliva [26,32,33].

Based on these advantages, we hypothesized that GG can be a therapeutic option as a delivery system for antifungals in the oral cavity. Therefore, in this study, we employed GG in different formulations to develop a drug delivery system for CAPE, evaluating its antifungal activity against *C. albicans* and efficacy in the treatment of denture stomatitis in an animal model.

## 2. Materials and Methods

### 2.1. Preparation of CAPE-GG Formulations

**Figure 1:**
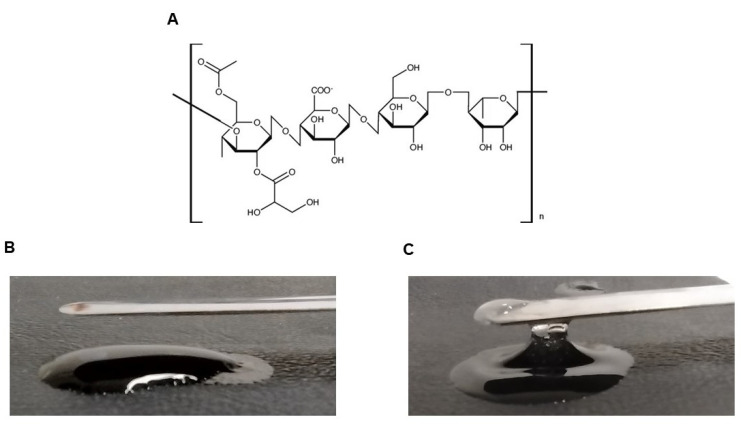
(**A**) Chemical structure of gellan gum [21]. (**B**) Aspect of GG-CAPE formulations at 0.6%. (**C**) Aspect of GG-CAPE formulations at 1%.

Initially, a solution of CAPE (Sigma-Aldrich, St. Louis, MO, USA) was prepared at 10 mg/mL diluted in dimethyl sulfoxide (DMSO; Sigma-Aldrich). After that, CAPE was diluted in sterile distilled water, and incorporated into GG (Sigma-Aldrich) at room temperature. Five different concentrations of GG (0.6; 0.7; 0.8; 0.9 and 1.0% *w*/*v*) were prepared. Calcium chloride (1 mM CaCl_2_; Sigma-Aldrich) was added to the CAPE-GG formulation to promote hydrogel gelation. The final concentration of CAPE in the GG formulation was 512 µg/mL. The chemical structure of GG and the characteristics of the CAPE-GG formulations at 0.6% and 1% are presented in Figure 1.

### 2.2. Ultraviolet-Visible (UV-Vis) Absorption Spectroscopy of CAPE, GG, and CAPE-GG Formulations

The absorption spectra in the UV–Vis region of CAPE, GG and CAPE-GG formulations were collected to confirm the incorporation of CAPE in GG, and to construct a calibration curve for determining the CAPE release kinetic from GG.

CAPE solution was diluted in concentrations ranging from 512.0 to 0.125 μg/mL, and 100 μL of each dilution was placed in 96-well UV-transparent plates (Corning, New York, NY, USA). The absorbance was measured in a spectrophotometer (Epoch, Biotek Instruments, Winooski, VT, USA) to scan from 260 to 700 nm, with 1 nm spacing. This same procedure was performed with GG and CAPE-GG formulations.

The CAPE solution demonstrated an absorbance peak at 325 nm and GG did not absorb in the same band. CAPE-GG formulations also presented an absorbance peak at 325 nm, confirming the incorporation of CAPE into GG. Based on these observations, 100 μL serial dilutions of CAPE solution were added in 96-well UV-transparent plates, and the absorbance were determined at 325 nm to construct a calibration curve (linear regression analysis) to determine the CAPE release from the formulations.

### 2.3. CAPE Release Kinetics

The release of CAPE from the CAPE-GG formulations was determined over different period of times. For this, 0.2 mm diameter biopsy bags (Thermo Fisher Scientific, Waltham, MA, USA) containing 1 mL of each CAPE-GG formulation (n = 5) was added into tubes with 9 mL of phosphate-buffered saline (PBS) and incubated at room temperature under agitation. Aliquots of 100 μL were removed at 10, 20, 30, 40, 50, 60, 90, 120, 150 and 180 min, placed in 96-well UV-transparent plates, and the absorbance was measured at 325 nm.

### 2.4. Microorganisms and Growth Conditions

One fluconazole-resistant clinical strain (*C. albicans* 70) and a reference strain (*C. albicans* SC5314) from the fungal collection of the Oral Microbiology and Immunology Laboratory at the Institute of Science and Technology of São José dos Campos/UNESP were used in this study [34]. The strains were cultured in yeast extract, peptone, dextrose broth (YPD; Difco, Detroit, MI, USA) for 24 h at 37 °C.

### 2.5. Antifungal Activity of CAPE and CAPE-GG Formulations by Determination of Minimum Inhibitory Concentration

Minimum inhibitory concentrations (MICs) were determined using the broth microdilution method according to the Clinical and Laboratory Standards Institute (CLSI), document M24-A4 [35]. CAPE-GG formulations and CAPE solution were diluted in the RPMI 1640 medium (Sigma-Aldrich) buffered to pH 7.0 with 0.165 M 3-(N-morpholino)propanesulfonic acid (MOPS; Sigma-Aldrich), and the 2-fold serial dilutions were added to 96-well plates. Aliquots of *C. albicans* suspensions were standardized, added to the wells at final concentrations of 0.5–2.5 × 10^3^ colony-forming units (CFU)/mL, and the plates were incubated at 37 °C for 48 h. Growth (RPMI + *C. albicans* strain) and sterile (pure RPMI) controls were included. The MIC was determined visually as the lowest concentration capable of inhibiting 100% of fungal growth.

### 2.6. Preparation of C. albicans Suspension for the Planktonic and Biofilm Assays

*C. albicans* strains were grown on Sabouraud dextrose agar (SDA; Himedia, Mumbai, India) for 48 h at 37 °C, and then plated in Yeast Nitrogen Base broth (YNB, Himedia) with 100 μM glucose (Vetec, RJ, Brazil) for 24 h at 37 °C. The suspension was centrifuged at 5000 rpm for 10 min and washed twice with PBS. Then, the cell concentration in the suspension was standardized using a hemocytometer to obtain 10^3^ and 10^8^ cells/mL for the planktonic and biofilm assays, respectively [35,36].

### 2.7. Time-Kill Assay in Planktonic Cultures

In a 24-well plate, *C. albicans* planktonic cells (10^3^ cells/mL) were treated with CAPE-GG formulations at 0.6 and 1.0%. Not treated group was included. The plates were incubated at 37 °C under agitation, aliquots were removed at 2 and 12 h, and plated on SDA. After incubation at 37 °C for 24 h, the CFU were counted and fungal viability (CFU/mL) was determined [37].

### 2.8. Biofilm Assay

Biofilms of *C. albicans* were formed at the bottom of 24-well plates. *C. albicans* suspension (10^8^ cells/mL) was added to each well, and the plates were incubated for 90 min at 37 °C under agitation for initial adhesion. After this period, the cells were washed twice with PBS, and fresh YNB broth was added. The plates were incubated at 37 °C for 24 h, and the biofilms were treated with CAPE-GG formulations at 0.6 and 1.0% for 2 and 12 h. Not treated group was included. Then, the biofilms were disrupted using an ultrasonic homogenizer (Sonopuls HD 2200, Bandelin Electronic, Berlin, Germany) at 7 W for 30 s [36]. Serial dilutions were prepared and plated on SDA. The plates were incubated for 24 h at 37 °C to determine the CFU/mL.

### 2.9. Evaluation of Proteolytic Activity (Sap)

Planktonic cells (10^3^ cells/mL) of *C. albicans* were treated with CAPE-GG formulations at 0.6 and 1.0%. A group of not treated cells was also included. Then, 100 μL of the supernatant from each group was collected and transferred to 400 μL of 0.1 M sodium citrate buffer containing 1% bovine serum albumin (BSA; Sigma-Aldrich). The samples were incubated for 1 h at 37 °C, and proteolytic activity was stopped by adding 5% trichloroacetic acid (TCA), followed by incubating for 1 h at 4 °C. Then, the mixture was centrifuged, and the supernatant was collected and placed in 96-well UV-transparent plates. The absorbance was measured at 280 nm [38]. The sap proteolytic activity was evaluated at 2, 4, 6, 8, 10 and 12 h.

### 2.10. CAPE Cytotoxicity to Human Keratinocytes (HaCat) Cells

HaCat cells were cultivated in Dulbecco’s Modified Eagle Medium (DMEM) (LGC Biotechnology, Cotia, Brazil) supplemented with 10% fetal bovine serum (FBS) (Invitrogen, New York, NY, USA), and incubated at 37 °C at 5% CO_2_. Cells at 4 × 10^4^ cell/well was added to 96-well plates, incubated for 24 h for cellular adherence, and treated with CAPE at concentrations ranging to 512 to 1.0 µg/mL. A control group with non-treated cells was included. Then, 200 µL of MTT solution (Sigma-Aldrich; 0.5 mg/mL PBS) was added for each well and the plates were incubated for 1 h at 37 °C in the absence of light. The solution was removed, 200 µL of DMSO was added for 10 min at 37 °C under agitation. The absorbance of final solution was measured at 570 nm [39]. The percentage of viability was determined using the formula: % Viability = (OD Treated Group × 100)/Mean OD Control Group).

### 2.11. In Vivo Study in Rat Model of Denture Stomatitis

#### 2.11.1. Animals and Experimental Groups

Adult 90 days old male Wistar rats (*Rattus norvegicus*, Albinus, Wistar), weighing approximately 400 g, from the Central Animal Care Facility of UNESP (Botucatu, SP, Brazil) were used in the study. This study was approved by the Ethics Committee on the Use of Animals of the ICT/UNESP (CEUA-ICT/UNESP, protocol number 03/2017), following the Ethical Principles for Animal Experimentation from the Brazilian College of Animal Experimentation (COBEA).

The animals were divided into the following experimental groups: animals with denture stomatitis and not treated (NT group, n = 5); animals with denture stomatitis treated with CAPE solution (CAPE group, n = 5); and animals with denture stomatitis treated with CAPE-GG formulation at 1.0% containing a final CAPE concentration of 15 mg/Kg (CAPE-GG group, n = 5).

Denture stomatitis was induced in rats by treatment with antibiotic therapy and the installation of an intraoral palatal device, according to the methodology proposed by Moraes et al. [40] with some modifications. Seven days prior to palatal device application, oxytetracycline hydrochloride with benzethonium hydrochloride (0.83 mg/mL, Terramycin^®^ in soluble powder with antigerm 77, Zoetis) was administered in the drinking water to reduce bacterial colonization in the oral cavity. The animals were fed with a pasty diet during 3 days before the device application and monitored daily.

#### 2.11.2. Preparation of Palatal Devices

**Figure 2:**
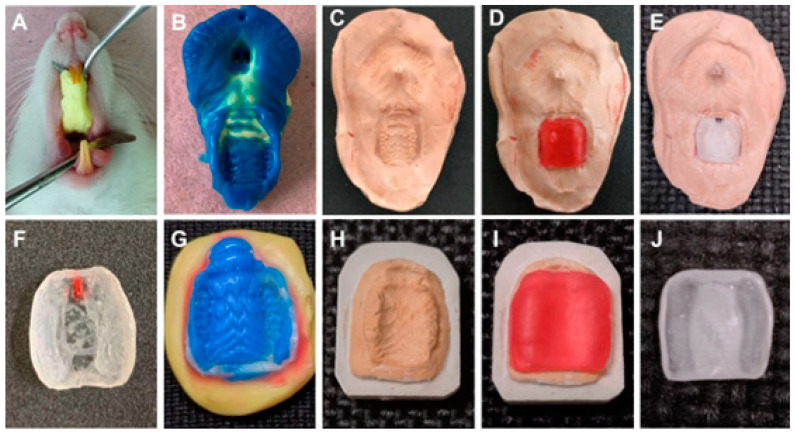
Preparation steps for palatal devices. (**A**) Impression with putty silicone C. (**B**) Mold obtained after impression with low-viscosity silicone C. (**C**) Preliminary model obtained. (**D**) Waxed model. (**E**) Polymerized custom tray. (**F**) Finished custom tray. (**G**) Impression with low-viscosity silicone C. (**H**) Obtained type IV stone working cast. (**I**) Waxed model. (**J**) Finalized palatal device.

Palatal devices were prepared according to the methodology proposed by Moraes et al. [40] with some modifications. The intraoral procedures were performed under general anesthesia by intraperitoneal injection with 100 mg/kg of 10% ketamine (Cetamin; Syntec do Brasil Ltda., São Paulo, Brazil) and 10 mg/kg of 2% xylazine (Xilazin; Syntec do Brasil Ltda., São Paulo, Brazil). A preliminary impression was made using putty and low-viscosity silicone C (Optosil and Xantopren; Heraeus Kulzer, Hanau, Germany) (Figure 2A,B), cast with type IV stone (Gesso Rio, Rio de Janeiro, Brazil) and waxed to a 1.5 mm thickness (Figure 2C,D).

A custom tray was fabricated in acrylic resin (Artigos Odontológicos Clássico, Campo Limpo Paulista, Brazil), polymerized by microwave radiation (Continental AW-30; Electrolux, Stockholm, Sweden) following manufacturers’ recommendations: 10% power/20 min, 60% power/5 min. Then, the tray was finished and 0.5 mm of relive at the teeth region was made. Polishing was performed with a rubber kit (KG Sorensen, Cotia, Brazil) (Figure 2E,F). Subsequently, a secondary impression with low-viscosity silicone C (Xantopren; Heraeus Kulzer) (Figure 2G) was made to obtain a working cast with type IV stone (Gesso Rio, Rio de Janeiro, Brazil). The palatal device was prepared (Figure 2H,I), and polished as previously described to obtain a custom tray (Figure 2J).

All devices were disinfected with chlorhexidine 2 (Rioquímica, São José do Rio Preto, São Paulo) and sterilized by exposure to ultraviolet radiation for 15 min on each face. Then, sterile swabs were rubbed on the devices faces, immersed in sterile saline, homogenized, and an aliquot of this solution was plated on Brain Heart Infusion (BHI, Himedia, Mumbai, India) agar. Microbial growth was evaluated after 48 h of incubation at 37 °C. The absence of microbial growth indicated the sterility of the dental devices.

#### 2.11.3. Standardization of *C. albicans* Suspension and Biofilm Formation on Palatal Devices

*C. albicans* 70 was grown on SDA for 48 h at 37 °C prior to assay. After this period, colonies of *C. albicans* were transferred to YPD and kept at 37 °C, at 75 rpm for 24 h. The fungal suspension was standardized at 1 × 10^8^ cells/mL [41].

The palatal devices previously sterilized were subjected to biofilm formation. The devices were placed in 24-well plates with 2 mL of *C. albicans* suspension in YNB with 100 μM glucose under agitation (75 rpm) for 90 min at 37 °C, for pre-adhesion. Then, the palatal devices were washed twice [42], placed in wells containing 2 mL of fresh YNB broth and maintained under agitation (75 rpm) at 37 °C for 24 h for biofilm formation.

#### 2.11.4. Installation of Palatal Devices in the Oral Cavity of Animals

The animals were anesthetized intraperitoneally with 10% ketamine at 100 mg/kg and 2% xylazine at 10 mg/kg. To verify the previous presence of *Candida* in the oral cavity of animals, samples were collected using a sterile swab with physiological solution, and the swab content was seeded in CHROMagar™ *Candida* (Difco) and incubated at 37 °C for 48 h.

After that, a swab soaked with the standardized suspension of *C. albicans* was passed over the entire palate of the animal. Then, 10 µL of CAPE solution or CAPE-GG formulation at 1.0% were added to the palatal devices covered by *C. albicans* biofilm. Then, the palatal devices were fixed using the RelyX Arc dual adhesive resin cement (3M ESPE, Maplewood, MN, USA), proportioned and manipulated according to the manufacturer’s recommendation. The animals remained with the palatal device for 48 h. After this period, the animals were euthanized with an overdose of 10% ketamine (300–400 mg/kg) associated with 2% xylazine (30–40 mg/kg).

#### 2.11.5. Fungal Burden and Histological Analysis

The development of the fungal infection was monitored by counting viable *C. albicans* (CFU/mL) cells and histological analysis. For fungal burden analysis, the palatal devices were carefully removed from the oral cavity, and a sample of the animals’ palate was collected using a sterile swab, which were immediately placed in saline solution. Each suspension was plated in CHROMagar™ *Candida* and SDA with chloramphenicol (0.1 mg/mL; Vixmicina, São Paulo, Brazil), followed by incubation at 37 °C for 48 h to determine the fungal burden (CFU/mL).

For the histological analysis, the hard palate was removed, cut into two sagittal portions, and fixed in 10% buffered formalin for 1 week. Next, the palatal tissues were processed by paraffin embedding and semi-serial sections of 5 µm thick were obtained. The cuts were stained with hematoxylin and eosin (H&E) and periodic acid-Schiff (PAS). The evaluation of the histological sections and photomicrographs were performed using an Axioplan 2 microscope and the Axiovision Rel 4.7 image acquisition program. Changes in the epithelial tissue were observed as characteristics of an inflammatory infiltrate, and the presence of yeasts and hyphae.

### 2.12. Statistical Analysis

Data were analyzed in the Prism 6 software (GrapPad Inc., San Diego, CA, USA) using Analysis of Variance (ANOVA), followed by the Tukey’s test. Values of *p* < 0.05 were considered statistically significant.

## 3. Results

### 3.1. Gellan Formulations Were Able to Incorporate CAPE and to Provide a Controlled Release

**Figure 3:**
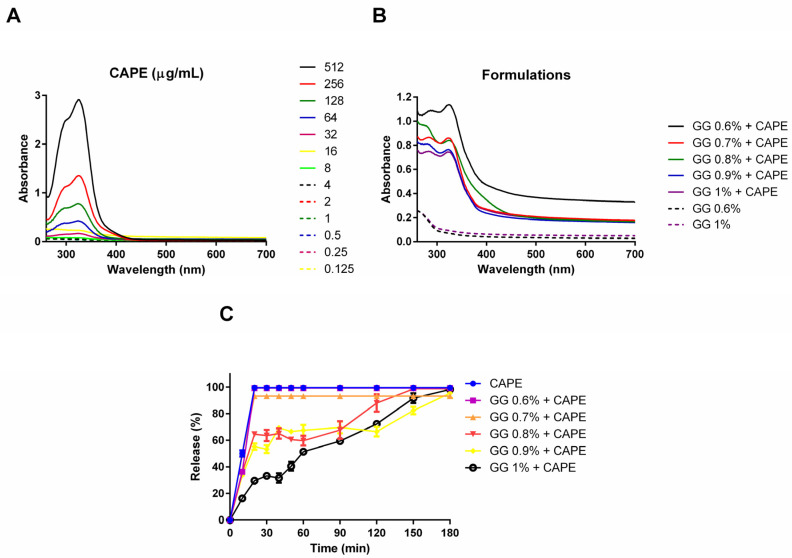
CAPE was incorporated into gellan formulations and released over time. Determination of optical density (OD) by ultraviolet and visible absorption spectroscopy: (**A**) CAPE solution in concentrations varying from 0.125 to 512 µg/mL; (**B**) CAPE-GG formulations of 0.6 to 1.0% (wt/vol); (**C**) Percentage and standard-deviation of CAPE release from GG formulations (0.6 to 1.0% wt/vol).

In the analysis of UV–Vis absorption spectroscopy, the CAPE solution exhibited an absorption band of 325 nm at concentrations ranging from 256 to 16 µg/mL. Absorption bands were not observed at concentrations below 8 µg/mL (Figure 3A). GG alone had absorption band close to 260 nm, while CAPE-GG formulations (0.6 to 1.0% wt/vol) showed an absorption band of 325 nm, indicating the incorporation of CAPE into gellan formulations (Figure 3B). In relation to release kinetics, CAPE-GG formulations at 0.6 and 0.7% exhibited a peak release near to 100% in 10 and 20 min, respectively. A slower and controlled release was found in the CAPE-GG formulations at 0.8, 0.9 and 1.0%. However, before 60 min, CAPE-GG formulations at 0.8% and 0.9% showed a faster release. Then, the CAPE-GG formulation at 1.0% had the slowest and most controlled release rate, with 100% CAPE releasing over 180 min (Figure 3C).

### 3.2. CAPE-GG Formulations Exhibited Antifungal Activity against C. albicans

CAPE solution inhibited *C. albicans* with MIC values ranging from 32 to 64 μg/mL. CAPE-GG formulations presented different MIC values according to the GG concentration, that ranged from 128 to higher than 512 μg/mL of CAPE. Antifungal activity of CAPE was reduced as the concentration of GG increased (Table 1).

### 3.3. CAPE-GG Formulations Had Effect against Planktonic Cells, Biofilms and Enzymatic Activity of C. albicans

**Figure 4:**
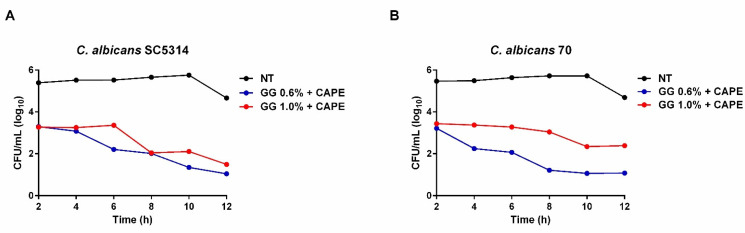
CAPE-GG formulations decreased the viability of *C. albicans* planktonic cells. (**A**,**B**) Mean of CFU/mL of *C. albicans* SC5314 and *C. albicans* 70 in planktonic cells not treated (NT) and treated with CAPE-GG formulations (0.6 or 1.0%) in different times of observation.

In planktonic cultures, CAPE incorporated in GG formulations decreased the viability of the *C. albicans* SC5314 reference strain. Treatment with both CAPE-GG formulations of 0.6 and 1.0% decreased the fungal viability by 2 log_10_ (CFU) at 2 h when compared to NT group. The reduction reached 3.6 log_10_ (CFU) at 8 h and approximately 4.0 log_10_ (CFU) at 12 h after the treatment with CAPE-GG formulations (Figure 4A). Furthermore, CAPE-GG formulations were able to reduce the viability of *C. albicans* 70, a fluconazole-resistant clinical strain. CAPE-GG formulations of 0.6 and 1.0% rapidly decreased the fungal viability by 2 log_10_(CFU) at 2 h. After 10 h, the fungal reduction reached 4.6 log_10_(CFU) for CAPE-GG 0.6% and 3.3 log_10_ for CAPE-GG 1.0%. At 12 h, the viability of *C. albicans* 70 was reduced by 3.6 and 2.3 log_10_(CFU) for 0.6 and 1.0%, respectively (Figure 4B).

**Figure 5:**
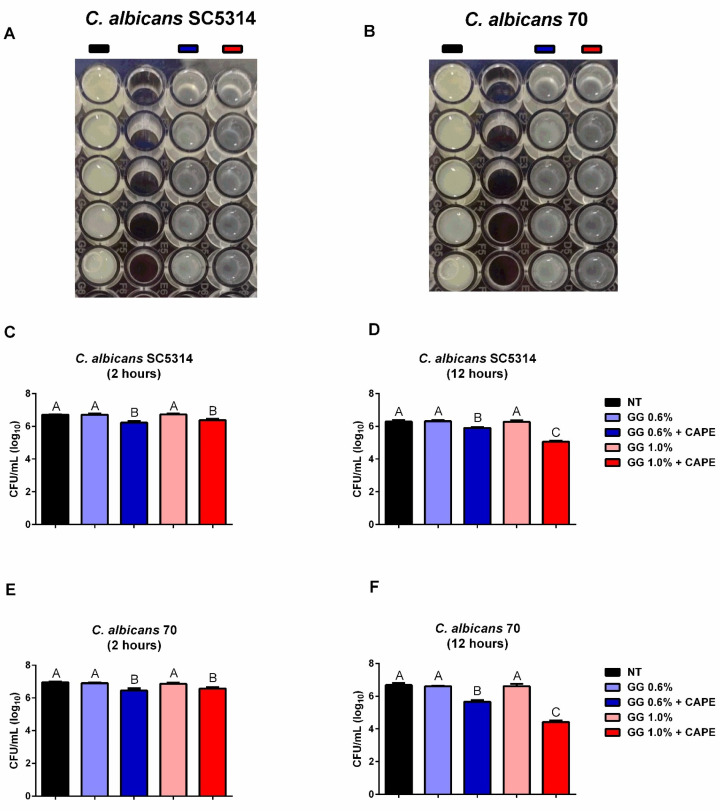
CAPE-GG formulations decreased the viability of *C. albicans* biofilms. (**A**,**B**) Aspects of biofilms of *C. albicans* SC5314 and 70 not treated (NT) and treated with CAPE-GG formulations (0.6 or 1.0%) after 12 h. (**C**,**D**) Mean and standard deviation of CFU/mL of *C. albicans* SC5314 and *C. albicans* 70 in biofilms not treated (NT) and treated with CAPE-GG formulations (0.6 or 1.0%) in the observation time of 2 h. (**E**,**F**) Mean and standard deviation of CFU/mL of *C. albicans* SC5314 and *C. albicans* 70 in biofilms not treated (NT) and treated with CAPE-GG formulations (0.6 or 1.0%) in the observation time of 12 h. Groups with statistically significant differences are represented by different letters (*p* < 0.05).

In addition, CAPE-GG formulations of 0.6 and 1.0% were able to decrease the viability of biofilms within 2 and 12 h after the treatments (Figure 5A,B). The treatment of *C. albicans* SC5314 reduced the biofilm cells by approximately 0.3 and 0.4 log_10_(CFU) at 2 h when compared to the NT group (*p* < 0.001) (Figure 5C). After 12 h, these reduction values were maintained for CAPE-GG 0.6% (0.3 log_10_ CFU) and accentuated for CAPE-GG 1.0% (1.25 log_10_ CFU, *p* < 0.001) (Figure 5D). CAPE-GG formulations were also effective in decreasing the biofilms of *C. albicans* 70, causing reductions of 0.3 and 0.5 log_10_(CFU) in relation to the NT group at 2 h (*p* < 0.001) (Figure 5E). For the time of 12 h, the treatment with CAPE-GG 0.6% reduced the biofilm viability by 1.0 log_10_(CFU), while the formulation of 1.0% caused a decrease of 2.1 log_10_(CFU) (*p* < 0.001) (Figure 5F). Thus, the CAPE-GG formulation of 1.0% caused a higher inhibition (*p* < 0.0001) than formulation of 0.6%, suggesting that a prolonged release of CAPE contributed to the reduction of biofilm viability.

**Figure 6:**
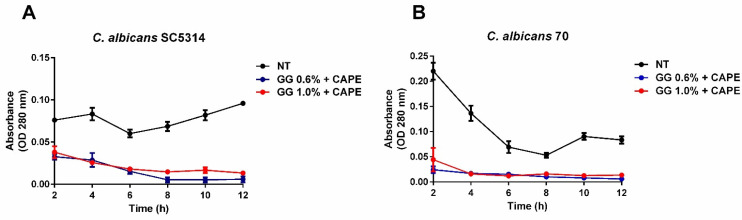
CAPE-GG formulations reduced the proteolytic activity of *C. albicans*. (**A**,**B**) Mean and standard deviation of optical density (OD) values obtained for *C. albicans* SC5314 and *C. albicans* 70 non-treated or treated with CAPE-GG formulations of 0.6 and 1.0%. Statistically significant difference was found between the non-treated group and CAPE-GG group for each time (*p* < 0.05).

The proteolytic activity for both *C. albicans* strains (SC5314 and 70) was also significantly reduced after 2 h of the treatments with CAPE-GG formulations (0.6 and 1.0%). The proteolytic activity remained inhibited until the end of the experiment (12 h) compared to non-treated control group (Figure 6A,B). These results suggested that the CAPE-GG formulations were able to act on virulence factors of *C. albicans*.

### 3.4. CAPE Was Not Cytotoxic to Keratinocytes

**Figure 7:**
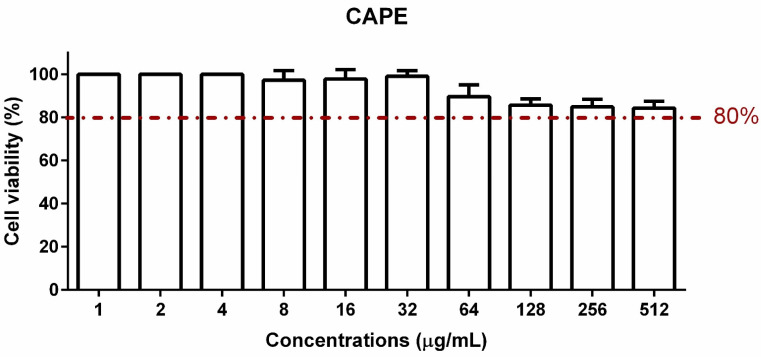
CAPE maintained viability of human cells. Percentage and standard deviation of cell viability (HaCat cells) treated with CAPE solution at concentrations ranging from 1 to 512 µg/mL normalized to untreated HaCat cells.

Treatment with CAPE for 24 h maintained 100% of keratinocytes viability at concentrations lower than 4 µg/mL. Reductions in cell viability were verified as the concentration of CAPE increased, reaching 10 and 15% reduction at 64 and 128 µg/mL, respectively. However, CAPE maintained at least 80% cell viability at the highest concentration used (512 µg/mL) (Figure 7).

### 3.5. GG-CAPE Formulation Reduced the Fungal Burden and Inflammation in Animal Model of Denture Stomatitis

**Figure 8:**
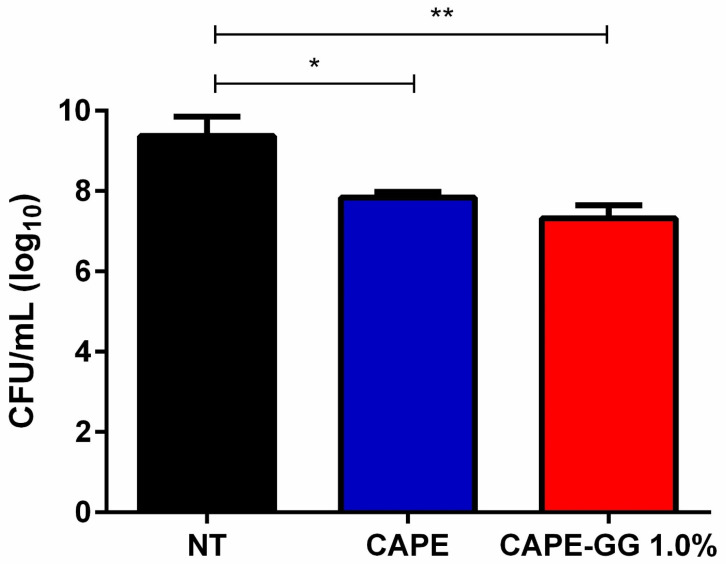
CAPE solution and CAPE-GG formulation reduced the fungal burden in animal model. Fungal burden (CFU/mL) of *C. albicans* in palate of rats for the groups: not treated (NT group), treated with CAPE solution (CAPE group) and treated with CAPE-GG formulation of 1.0% (CAPE-GG group). * (*p* < 0.05) statistically significant difference compared to NT group. ** (*p* < 0.01) statistically significant difference compared to NT group.

Treatment with CAPE solution and CAPE-GG formulation of 1.0% reduced the fungal burden compared to non-treated control group. The CFU/mL count recovered from the oral cavity of animals was 9.37 log_10_(CFU) for the control group, while it was 7.84 log_10_(CFU) for the treatment with CAPE solution (*p* = 0.0235) and 7.32 log_10_(CFU) for the treatment with CAPE-GG formulation (*p* = 0.0087) (Figure 8).

**Figure 9:**
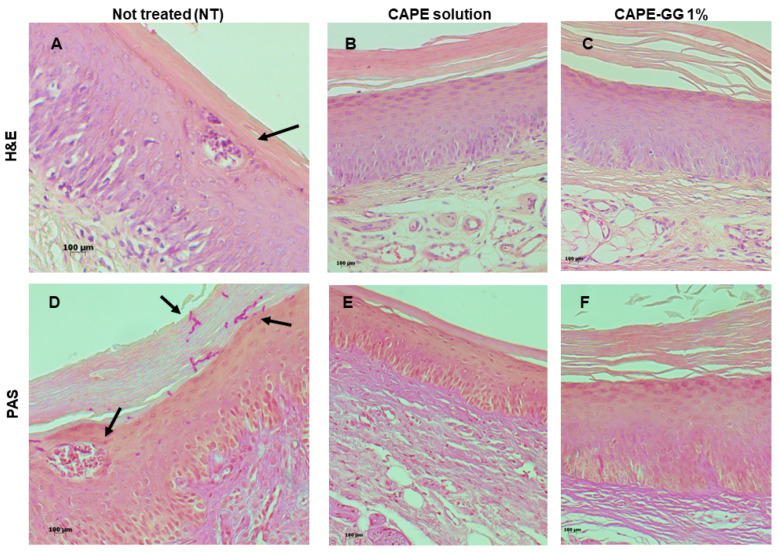
Histological analysis of the palate of rats with a palatal device and infected with *C. albicans*. Images of histological cuts stained by hematoxylin and eosin (H&E): (**A**) not treated group, (**B**) treated with CAPE solution, and (**C**) treated with CAPE-GG formulation of 1.0%. Images of histological cuts stained by periodic acid-Schiff (PAS): (**D**) not treated group, (**E**) treated with CAPE solution, and (**F**) treated with CAPE-GG formulation of 1.0%. In the NT group, black arrows indicate inflammatory cells and hyphae. CAPE: caffeic acid phenethyl ester; GG: gellan gum; NT: not treated.

In relation to histological analysis of palatal mucosa, the non-treated control group showed an infiltration of *Candida* hyphae in the keratin layer and the presence of inflammatory cells forming intraepithelial microabscesses, confirming the induction of the denture stomatitis model. Interestingly, CAPE and CAPE-GG formulation groups exhibited an epithelium with fewer fungal and inflammatory cells. In these treated groups, no hyphal infiltration and microabscesses were found. In all the three groups analyzed, no signs of tissue toxicity by CAPE or GG were observed (Figure 9).

## 4. Discussion

*Candida* infections are frequently found in the oral cavity, mainly in immunocompromised patients and dental prosthesis users. Since the treatments are limited to few therapeutic options with elevated relapse rates, new antifungal agents have been investigated. In the previous study of our group, we evaluated if caffeic acid phenethyl ester (CAPE) is active against *C. albicans* and experimental candidiasis. CAPE exhibited antifungal activity against planktonic cells and mature biofilms of *C. albicans.* Treatment with CAPE also prolonged survival of *Galleria mellonella* larvae infected by *C. albicans* and reduced fungal burden in a murine model of oral candidiasis in the tongue dorsum [15]. Although CAPE showed potential to be used in the treatment of candidiasis, its low viscosity in a liquid solution can reduce the retention time in the oral cavity, and consequently, compromise the antifungal activity. In addition, factors such as salivary flow, tongue movements and swallowing can limit the retention of the compounds on the mucosa and prosthetic surfaces [8]. To overcome these limitations, an interesting alternative can be the use of a drug delivery system to provide a sustained release of the antifungal compound at the target site [43,44]. Therefore, the proposal of the present study was to develop a delivery system for CAPE using gellan gum hydrogel (GG) and to evaluate its in vitro and in vivo effects against *C. albicans*. To the best of our knowledge, this is the first study that employed GG as carrier system for CAPE targeted to oral fungal infections.

Firstly, we studied the capacity of GG to incorporate CAPE using a spectroscopy analysis, in which CAPE solution exhibited absorption maximum at 325 nm. Bai et al. [45] found 330 nm as the maximum absorption of CAPE solution, but it is known that small variations may occur due to the difference in vehicle used for dilution [46]. On the other hand, the absorption peak of GG around 260 nm like the results of previous studies [47,48,49]. This absorption peak of GG can be attributed to the presence of glucuronic acid. Promisingly, CAPE-GG formulations of 0.6 to 1.0% (wt/vol) presented absorption maximum at 325 nm, confirming that CAPE was incorporated into GG hydrogel in all GG concentrations studied.

Then, we evaluated the release kinetic of CAPE from different GG formulations. The release of CAPE from the hydrogels was normalized to the total loading in the hydrogel. Early increased release was observed for the formulations at lower concentrations of GG (0.6 and 0.7%), while higher concentrations (0.8 to 1.0%) exhibited more prolonged release of CAPE (180 min). Indeed, higher polymer concentrations lead to more entanglements and crosslinks within the hydrogel, which can increase the migration time and timescales of drug release from hydrogels [50]. Furthermore, it is possible that the higher concentrations of GG enhanced the physical and chemical entrapment of CAPE, possibly due to intermolecular interactions [51]. However, this hypothesis requires further investigation.

Since GG was able to incorporate and release CAPE, we moved to study the antifungal activity of CAPE-GG formulations against *C. albicans,* comparing its results with the CAPE solution. In the assay, to determine the Minimum Inhibitory Concentrations (MIC), CAPE solution exhibited in vitro activity against *C. albicans* with MIC values ranging from 32 to 64 μg/mL. However, when CAPE was incorporated into GG, MIC values increased as the concentration of GG increased in the formulation. Zimmermann et al. [52] studied the anti-*Candida* effects of diphenyl diselenide-loaded nanocapsules free and incorporated into GG. Although the GG-incorporated nanocapsules exhibited antifungal activity against *C. albicans* and *C. glabrata*, higher MIC values were observed when compared to the free nanocapsules, as demonstrated in our study.

Based on these results, the CAPE-GG formulations of 0.6 and 1.0% were chosen for the subsequent assays. While the CAPE-GG at 0.6% showed accelerated and short release of CAPE, with reduced MIC values; the formulation of 1.0% provided controlled and prolonged release of CAPE related to higher MIC values. The ability of CAPE-GG at 1% to be released in a controlled time of up to 180 min is especially important for infections in certain anatomical sites, such as oral mucosa, in which salivary clearance can rapidly dilute and weaken the active compound [53]. The prolonged release capacity of CAPE-GG at 1% can increase the antifungal action time and improve the drug targeting. Consequently, CAPE can be more effective in causing damage to fungal cells, raising the possibility of applying these hydrogels to treat several buccal manifestations of *Candida* infections [54,55,56], including pseudomembranous, erythematous, and hyperplastic candidiasis.

In planktonic and biofilm assays, both CAPE-GG formulations of 0.6 and 1% reduced the viability of *C. albicans* cells at 2 and 12 h post-treatment. It is known that biofilms confer protection for *C. albicans* against the host immune system and antifungal therapy, making the biofilms 30 to 200 times more resistant to antimicrobial drugs when compared to planktonic cells [6,57]. Barros et al. [15] demonstrated that CAPE exhibited inhibitory effect in the expression of genes involved in maintenance, development, and maturation of biofilms, such as transcriptional regulators, adhesion, and filamentation genes. It is also suggested that CAPE induce programmed cell death in *C. albicans*, that consequently reduce the fungal viability and the biofilm formation [58,59].

Interestingly, CAPE-GG formulation of 1.0% exhibited a higher antibiofilm activity compared to CAPE-GG formulation of 0.6%. This result can be attributed to controlled and prolonged release of CAPE from the GG in formulation of 1.0% that possibly provided a higher penetration into biofilms. Drug diffusion through *C. albicans* biofilm is directly associated with its susceptibility to antifungal therapy, mainly due to polymeric substances of extracellular matrix [60]. Studies revealed that the concentration and release rate of compounds are important factors for their penetration on biofilms [61,62,63]. Al-Fattani and Douglas [61] analyzed the penetration of several antifungals (fluconazole, amphotericin B and voriconazole) into *Candida* biofilms, verifying that the drug reached the distal edges of biofilms only when it was used in concentrations substantially higher than the MIC values. Using a drug delivery system, Torabiardekani et al. [63] and Karami et al. [62] demonstrated that the encapsulation of essential oils in hydrogels provided their controlled and prolonged release for 270 min, which was associated with an enhanced activity against *C. albicans* biofilms. These results confirm that modern drug delivery systems based on hydrogels can assist by achieving better therapeutic effects with slower release process [13].

In addition, CAPE-GG formulations of 0.6 and 1% decreased the proteolytic activity of *C. albicans*. Proteolytic activity, an important virulence factor of *C. albicans*, is mediated mainly by saps that cleaves different peptides, including proteins related to host immune response [64,65]. Saps have a family of 10 genes (*Sap1* to *Sap10*) related to entering nutrients into the cell, blocking components of the complement system, releasing pro-inflammatory peptides, and adhesion to host epithelial cells [66,67,68]. Some studies reported that conventional antifungal therapy increased *Candida* proteolytic activity as a compensatory mechanism [69]. It was demonstrated that treatment with fluconazole and voriconazole increased the extracellular sap activity in fluconazole and voriconazole-resistant strains, probably due to the efflux mechanism that regulates sap transport [70,71]. Interestingly, here we found that CAPE-GG formulations reduced proteolytic activity of *C. albicans* for the reference strain and the clinical fluconazole-resistant strain.

Therefore, our results indicated that the CAPE released from GG formulations were able to reduce planktonic cells, biofilm, and even proteolytic activity of *C. albicans,* suggesting a multi-target mode of action. Then, we investigated the toxicity of CAPE in mammalian cells. CAPE was not cytotoxic to keratinocytes at 512 µg/mL, that was the higher concentration used. Similarly, Yin et al. [72] demonstrated that CAPE maintained human oral keratinocytes viability up to 160 µg/mL. Indeed, CAPE has been considered a pharmacologically safe compound, since no harmful effects toward hepatocytes, erythrocytes and other mammalian cells have been reported [73,74,75,76].

Finally, the results were expanded to an in vivo study using a denture stomatitis model in rats. With this model, we reproduced the in situ conditions that can interfere with the antifungal treatments, such as the presence of mucous membranes, teeth, prosthetic devices, salivary flow, pH variations, and immune response [40,77,78]. Even in the face of these conditions, CAPE-GG formulation of 1.0% and CAPE solution reduced the fungal cells (CFU/mL) in the palate when compared to untreated group, indicating that CAPE was released in vivo from the gellan. In addition, the CAPE released maintained its antifungal properties similarly to CAPE solution, confirming that GG did not alter the pharmacological properties of CAPE.

In addition to the counting of CFU/mL, the palatal mucosa was evaluated by histological analysis. Non-treated control group exhibited *C. albicans* cells adhered to mucosa surface, hyphae penetrating in the stratum corneum of epithelium, and neutrophilic micro abscesses, corroborating to the findings of previous studies of denture stomatitis model [79]. The groups treated with CAPE-GG formulation or CAPE solution presented a reduction in the presence of yeasts and inflammatory cells, and hyphal infiltration and micro abscesses were not found. It has been reported that CAPE has antioxidant properties by scavenging reactive oxygen species, suppressing lipid peroxidation, and reducing malondialdehyde production. Furthermore, CAPE stimulates the activity of antioxidant enzymes by enhancing superoxide dismutase, catalase, peroxidase, and glutathione levels [80,81,82]. CAPE also possess anti-inflammatory properties by inhibiting NF-κB, inflammatory cytokines, and activating Nrf2 signaling [82]. Altogether, these antioxidant, anti-inflammatory and antimicrobial properties [15] can explain the reduction of inflammation, filamentation and fungal burden observed after CAPE treatment.

Since denture stomatitis has high prevalence and recurrence [4,83], many other studies have evaluated drug delivery systems for prevention and treatment of this oral disease [79,84,85]. For this, Sultan et al. [79] developed an animal model of denture stomatitis using 3D digital imaging and printing technology to fabricate intraoral devices. Since this model can be easily digitally modified and optimized, it can be used to explore different therapeutic strategies. After establishing the intraoral device, the authors evaluated a peptide-based hydrogel as oral topical formulation for denture stomatitis. The prophylactic use of formulation was efficacious in reducing the fungal burden, as well as clinical resolution of the palatal mucosa. However, the formulation was ineffective in clearing preformed biofilms of infected dental devices.

Overall, our in vivo results showed that CAPE-GG formulations were a safe therapeutic strategy to decrease the fungal burden and inflammation of rats with denture stomatitis. However, more in-depth studies and refined animal models are required to optimize the advantages of controlled release of CAPE-GG formulations, and consequently, to maximize its therapeutic efficacy. In future studies, special focus must be given to physical, mechanical, and chemical properties of the CAPE-GG formulations, exploring different analytical techniques, such as rheological measurements, sol-gel analysis, texture analysis, tensile testing, confocal laser scanning microscopy, scanning electron microscopy, atomic force microscopy, and nuclear magnetic resonance [21,86].

## 5. Conclusions

In summary, we demonstrated that GG was able to incorporate CAPE and provide a controlled release, exerting antifungal activity against planktonic cells and biofilms of *C. albicans*. Furthermore, CAPE-GG formulations were able to reduce the proteolytic activity of *C. albicans*, an important fungal virulence factor. Interestingly, these effects were also observed in a fluconazole-resistant *C. albicans* strain. In addition, CAPE did not show toxicity in vitro in human keratinocytes and was safe in animal models. The treatment with CAPE-GG formulations was able to reduce the fungal burden and inflammation in denture stomatitis in rats, reinforcing its in vivo antifungal activity.

## Figures and Tables

**Table 1:** Minimum inhibitory concentration (MIC) of CAPE and CAPE in gellan gum (GG) formulations at 0.6, 0.7, 0.8, 0.9 and 1.0% against *Candida albicans* strains.

Strain	MIC (µg/mL)					
	CAPE	GG 0.6% + CAPE	GG 0.7% + CAPE	GG 0.8% + CAPE	GG 0.9% + CAPE	GG 1.0% + CAPE
*C. albicans* SC5314	64	512	512	>512	>512	>512
*C. albicans* 70	32	128	128	256	256	512
Range	32–64	128–512	128–512	265–> 512	256–> 512	512–> 512

## Data Availability

Data will be made available on request.
